# Genome-Assisted Prediction of Quantitative Traits Using the R Package *sommer*

**DOI:** 10.1371/journal.pone.0156744

**Published:** 2016-06-06

**Authors:** Giovanny Covarrubias-Pazaran

**Affiliations:** Department of Horticulture, University of Wisconsin, Madison, Wisconsin, Unites States of America; Institute of Genetics and Developmental Biology, CHINA

## Abstract

Most traits of agronomic importance are quantitative in nature, and genetic markers have been used for decades to dissect such traits. Recently, genomic selection has earned attention as next generation sequencing technologies became feasible for major and minor crops. Mixed models have become a key tool for fitting genomic selection models, but most current genomic selection software can only include a single variance component other than the error, making hybrid prediction using additive, dominance and epistatic effects unfeasible for species displaying heterotic effects. Moreover, Likelihood-based software for fitting mixed models with multiple random effects that allows the user to specify the variance-covariance structure of random effects has not been fully exploited. A new open-source R package called *sommer* is presented to facilitate the use of mixed models for genomic selection and hybrid prediction purposes using more than one variance component and allowing specification of covariance structures. The use of *sommer* for genomic prediction is demonstrated through several examples using maize and wheat genotypic and phenotypic data. At its core, the program contains three algorithms for estimating variance components: Average information (AI), Expectation-Maximization (EM) and Efficient Mixed Model Association (EMMA). Kernels for calculating the additive, dominance and epistatic relationship matrices are included, along with other useful functions for genomic analysis. Results from *sommer* were comparable to other software, but the analysis was faster than Bayesian counterparts in the magnitude of hours to days. In addition, ability to deal with missing data, combined with greater flexibility and speed than other REML-based software was achieved by putting together some of the most efficient algorithms to fit models in a gentle environment such as R.

## Introduction

With next generation sequencing technologies (NGS) becoming cheaper and consequently more feasible for all crops, huge genomic data sets have become available to help during selection and decision making in plant breeding programs [1,2]. The idea of using genetic markers to accelerate and improve plant and animal breeding systems originated with Sax in 1923 who first reported an association of a simply inherited genetic marker with a quantitative trait in plants [3,4]. On the other hand, the development of statistical tools for breeding purposes, particularly, Henderson’s mixed model equations in 1949 (not published until 1963 with the help of Searle), gave rise in animal breeding to kinship-based selection, breeding value estimation, and prediction of breeding materials [5–7]. More recently, genetic markers have been exploited in plant breeding to detect quantitative trait loci (QTL) for marker assisted selection (MAS). However, after decades of QTL studies, the real application and value of the QTL paradigm in plant breeding has been questioned [8,9].

With the advent of inexpensive and high-throughput genotyping technologies in the last decade, a new plant and animal breeding selection paradigm called genomic selection has emerged [4]. Genomic selection allows the prediction of the phenotypes of individuals based on known marker effects or genetic relationships (kinship-based), and in plants it has been used for predicting trait performance of hybrids and unrealized crosses. One of the first methods proposed for genomic selection was a statistical method called ridge regression (RR), where the ridge parameter (λ) can be observed in a mixed model framework as the σ^2^_e_ / σ^2^_u_ ratio between the residual and random effect variances. This can be applied in the genomic context where σ^2^_u_ is the genetic variance and best linear unbiased predictor (BLUP) can be interpreted as the genomic estimated breeding values (GEBV), where the random effect refers to genotype effects and the variance-covariance structure is the additive or genomic relationship matrix (**A** or **A**_g_). The genetic variance can also be interpreted in terms of marker effects in the form of marker-based BLUPs [10–13].

The use of mixed models to estimate breeding and genetic values can be generalized to more complex scenarios. Mixed models can be used to address general and specific combining abilities in hybrid populations. In particular, they can be used to predict the performance of unrealized crosses, such as single cross hybrids in species which commonly display additive and dominance (heterosis) effects [7,14]. These effects, also called general and specific combining abilities (GCA and SCA, respectively), can be dissected in a mixed model as random effects with a particular variance-covariance structure (**G**) and with distribution:

$$\left[\begin{array}{c} y\\ u\\ \varepsilon \end{array}\right]∼\left(\left[\begin{array}{c} X\beta \\ 0\\ 0 \end{array}\right]\;,\;\left[\begin{array}{ccc} ZGZ^{\prime }+R & ZG & R\\ GZ\prime & G & 0\\ R & 0 & R \end{array}\right]\;\right)\;\text{being}\;G=\left[\begin{array}{cccc} K_{1}\sigma_{u1}^{2} & 0 & 0 & 0\\ 0 & K_{2}\sigma_{u2}^{2} & 0 & 0\\ 0 & 0 & \ldots & 0\\ 0 & 0 & 0 & K_{j}\sigma_{uj}^{2} \end{array}\right]$$

Here, **X** and **Z** are incidence matrices for fixed and random effects respectively, **R** = **I**σ^2^_e_, **K**_i_ is the variance covariance structure for the i^th^ random effect and σ^2^_ui_ is the variance component for the i^th^ random effect. Such covariance structures in a general mixed model are usually unknown, but in genomic selection theory such covariance structures are expressed as relationships among individuals, estimated by an additive, genomic, or other type of relationship matrix [13]. Despite all the molecular and statistical advances that allow genomic selection, there is few open source genomic selection or mixed model software that allows the modeling of several variance components at a time and particularly the modeling of SCA effects by likelihood methods, such as some popular R packages; regress, and EMMREML [13,15–19]. The purpose of this paper is to describe the R package *sommer* (solving mixed model equations in R), an open-source REML-based package that can handle more than one variance component, and at the same time allows for flexible specification of variance-covariance structures of random effects and compare it to popular Bayesian and Likelihood-based software. *Sommer* is especially useful for hybrid prediction of species displaying strong heterotic or specific combining ability effects. The package relies on three algorithms based on maximum likelihood (ML) and restricted maximum likelihood (REML); efficient mixed model association (EMMA) [20], direct average information (AI) [21,22], and expectation maximization (EM) [23,24]. In addition, *sommer* includes other kernels for calculating additive, dominance and epistatic relationship matrices [25] and perform genome wide association studies (GWAS) (the software can be found and downloaded at https://cran.rstudio.com/web/packages/sommer/ [verified 10 May 2016]). The key features of the package are demonstrated using wheat data (*Triticum aestivum* L.) for genomic prediction of species displaying small or null heterotic effects where only the additive kernel is required (a single random effect), and prediction of single-cross maize hybrids (*Zea mays* L.), which require the use of additive and dominance kernels (multiple random effects), and can be extended to any species displaying heterotic effects.

## Materials and Methods

### Algorithms

The *sommer* package solves the mixed model equations proposed by Henderson [6], and it has been implemented to work with incidence matrices and known variance covariance matrices for each random effect through the use of the *mmer* function and a ASReml-type version named *mmer2*. If an incidence or a variance-covariance matrix is omitted, the software assumes an identity matrix. Currently, three algorithms for variance component estimation are supported; efficient mixed model association (EMMA) [18], average information (AI) [21,22], and expectation maximization (EM) [23,24]. The EMMA method is useful when only one variance component other than the error variance component (σ^2^_e_) is estimated [25]. When more than one variance component needs to be estimated, the AI and EM algorithms should be used. The AI algorithm is the default, similar to other commercial software such as ASReml [21].

### Genomic breeding value estimation in a wheat population

We performed genomic breeding value estimation (GEBV) and hybrid prediction with wheat data, and the results were compared to other genomic selection and mixed model software, including rrBLUP [13], ASReml [21], regress (used by synbreed as well) [17,18], EMMREML [19], MCMCglmm [15], and BGLR [16]. We used the wheat data contained in the R package BGLR consisting of 599 inbred lines genotyped with 1279 diversity array technology (DArT) markers [16]. Phenotypic data consisted of grain yield (GY) for the 599 lines from the historical CIMMYT's Global Wheat Program evaluated in four mega-environments.

From the 599 wheat lines, 179,101 distinct single crosses can be performed. Kinship-based BLUP prediction for the 599 lines were obtained using rrBLUP (ridge regression), ASReml (average information), regress (Newton-Raphson), EMMREML (modified EMMA), BGLR (using the Reproducing kernel Hilbert space [RKHS] kernel), MCMCglmm (Gibbs sampling) and the three algorithms implemented in *sommer* (AI, EM, and EMMA). Similarity among BLUPs using all software was performed in R and displayed in tables and figures [26]. The genomic estimated breeding values (GEBV) for each of the 599 inbred lines was used to predict the performance of possible crosses as the average among the breeding value of the parental lines. The mixed model fitted has the form:

y=Xβ+Zu+ε

with variance:

$$V(y)=V(Zu+\varepsilon )={ZGZ}^{\prime }+R$$

and the mixed model equations for this model are:

$${\left[\begin{array}{cc} X\prime R^{-1}X & X\prime R^{-1}Z\\ Z\prime R^{-1}X & Z^{\prime }R^{-1}Z+G^{-1} \end{array}\right]}^{-1}\left[\begin{array}{c} X\prime R^{-1}y\\ Z\prime R^{-1}y \end{array}\right]=\left[\begin{array}{c} \beta \\ u \end{array}\right]$$

Here, **G** = **K**σ^2^_u_, is the variance covariance matrix of the random effect u, from a multivariate normal distribution u ~ MVN(0, **K**σ^2^_u_), K being, in the genomics context, the additive or genomic relationship matrix (**A** or **A**_g_). **X** and **Z** are incidence matrices for fixed and random effects respectively, and **R** is the matrix for residuals (here **I**σ^2^_e_). A mixed model with a single variance component other than the error (σ^2^_e_) can be used to estimate the genetic variance (σ^2^_u_) along with genotype BLUPs to exploit the genetic relationships between individuals coded in **K** (**A**). The genomic relationship matrix was constructed according to VanRaden where **K** = **ZZ**’/2Σp_i_(1-p_i_) [27]. Genotype BLUPs were calculated and considered equal to the GEBV and these were used to predict the performance of the 179,101 possible crosses as the average of parental genomic breeding values. We fitted this model using the *sommer* package by specifying the incidence and variance-covariance matrices and using the three algorithms implemented (AI, EM, EMMA). In addition, a five-fold cross validation was performed to calculate the predictive correlation for grain yield in the four mega environments available for the wheat data using the *sommer* package. In addition, heritability was estimated as h^2^ = σ^2^_u_ / σ^2^_u_ + σ^2^_e_.

### Single cross hybrid prediction in corn

Genotypic data was simulated consisting of 511 SNP markers in 40 inbred lines belonging to two heterotic groups (20 in each). Phenotypic data was simulated consisting of grain yield (GY) and plant height (PH) for the 40 parents and 100 out the 400 possible hybrids produced from the single-cross of both heterotic groups allowing for heterosis. Genotypes of the 40 parents were used to estimate the genomic relationship matrices as **K** = **ZZ**’/2Σp_i_(1-p_i_) [27] for both heterotic groups (**K**_1_ and **K**_2_), and the genomic relationship matrix for the 400 possible hybrids was obtained as the Kronecker product of the parental genomic relationship matrices **K**_1_ ⊗ **K**_2_ (**K**_3_). Given that the phenotypic data for the possible crosses was not masked, the hybrids were predicted by estimating the BLUPs for general combining abilities in males and females (GCA_female_, GCA_male_) and specific combining abilities (SCA) of crosses along with their variance components (σ^2^_GCA1_, σ^2^_GCA2_, σ^2^_SCA_). The model has the form:

$$y=X\beta +Z_{1}u_{GCA1}+Z_{2}u_{GCA2}+Z_{3}u_{SCA}+\varepsilon$$

The mixed model equations for this model are:

$${\left[\begin{array}{cccc} X\prime \;R^{-1}X & X\prime \;R^{-1}Z_{1} & X\prime \;R^{-1}Z_{2} & X\prime \;R^{-1}Z_{3}\\ Z_{1}\prime \;R^{-1}X & Z_{1}\prime \;R^{-1}Z_{1}+G_{1}^{-1} & Z_{1}\prime \;R^{-1}Z_{2} & Z_{1}\prime \;R^{-1}Z_{3}\\ Z_{2}\prime \;R^{-1}X & Z_{2}\prime \;R^{-1}Z_{1} & Z_{2}\prime \;R^{-1}Z_{2}+G_{2}^{-1} & Z_{2}\prime \;R^{-1}Z_{3}\\ Z_{3}\prime \;R^{-1}X & Z_{3}\prime \;R^{-1}Z_{1} & Z_{3}\prime \;R^{-1}Z_{2} & Z_{3}\prime \;R^{-1}Z_{3}+G_{3}^{-1} \end{array}\right]}^{-1}\left[\begin{array}{c} X\prime \;R^{-1}y\\ Z_{1}\prime \;R^{-1}y\\ Z_{2}\prime \;R^{-1}y\\ Z_{3}\prime \;R^{-1}y \end{array}\right]=\left[\begin{array}{c} \beta \\ u_{GCA1}\\ u_{GCA2}\\ u_{SCA} \end{array}\right]$$

where β is the vector of fixed effects, u_GCA1_, u_GCA2_, u_SCA_ are the BLUPs for GCA_female_, GCA_male_, and SCA effects, **X** and **Z**s are incidence matrices for fixed and random effects respectively, **R** is the matrix for residuals (here **I**σ^2^_e_), and **G**^-1^_1_, **G**^-1^_2_, **G**^-1^_3_ are the inverse of the variance-covariance matrices for random effects. The BLUPs u_GCA1_, u_GCA2_, u_SCA_ were used to predict the rest of the single-crosses as the sum of their respective GCA and SCA effects.

We fitted this model using the *sommer* package by specifying the incidence and variance-covariance matrices and using the AI and EM algorithms, given that EMMA method cannot estimate more than one variance component. The model could not be implemented in rrBLUP which is also limited to a single variance component. In the BGLR package the Reproducing kernel Hilbert space [RKHS] kernel was used, in ASReml and MCMCglmm the ‘ginverse’ argument was used to specify the variance-covariance structures, and in the regress package the multiple random effects model using the **ZKZ**’ kernel was fitted. EMMREML uses a similar syntax than *sommer*. Results from other software were compared with *sommer*. In addition, a five-fold cross validation was performed to calculate the prediction accuracy for plant height and grain yield in this population.

In order to show the advantage of fitting a model including dominance (SCA) compared to a pure additive models (GCA) with respect to the prediction ability for species displaying heterotic effects, two additional models were fitted including only GCA effects; 1) both parents having the same variance component and 2) each parent from a different heterotic group having a different variance component:

$$G=[\begin{array}{c} {K\sigma }_{u}^{2} \end{array}]\;and\;G=\left[\begin{array}{cc} K_{1}\sigma_{u1}^{2} & 0\\ 0 & K_{2}\sigma_{u2}^{2} \end{array}\right]$$

Models were compared with respect to their prediction ability after 500 runs of a five-fold cross validation for plant height and grain yield. Models were fitted using *sommer* with the default AI algorithm. In addition, heritability for both trait was estimated as; h^2^ = (σ^2^_GCA1_ + σ^2^_GCA2_) / (σ^2^_GCA1_ + σ^2^_GCA2_ + σ^2^_e_).

### Capabilities with big data sets and comparison with other software

In order to test the capabilities of *sommer* compared with other software, posterior analysis were performed with REML-based counterparts: rrBLUP, regress, ASReml, EMMREML, and Bayesian-based: BGLR (iterations = 13000, burn-in = 2000), and MCMCglmm (iterations = 13000, burn-in = 2000; default parameters). Such comparisons were performed using bigger data sets. We simulated phenotypic and genotypic data for 5000 individuals with 10000 markers for a single trait and single additive kernel, with heritability h^2^ = 0.5 and GEBVs were estimated. Computing time as a function of the population size (N) for the different ML/REML algorithms found across software packages for a single variance component scenario was recorded. We recorded elapsed times for population sizes from 500 to 5000 in intervals of 500 increments and plotted using R.

The phenotypic and genotypic data available from Technow et al. [28] was used to predict the genetic value (GV) of 10578 possible single cross hybrids from the cross of the Flint by Dent heterotic groups, which included additive and dominance effects (three variance components). The same model was fitted with *sommer* counterparts when possible for time and flexibility comparison purposes. Flint and Dent lines were genotyped with 35,432 SNP markers. Computing time as a function of the population size (N) was recorded for population sizes from 1000 to 8000 in intervals of 1000 increments using *sommer* and other REML-based software with ability to fit multiple random effects.

All genotypic and phenotypic information used in this research is freely accessible and can be found in the R package documentation. The maize data can be accessed as data(cornHybrid), data(wheatLines), and data(Technow_data). The script for all analysis can be found in S1 File.

## Results and Discussion

At the core of the *sommer* package is the function ‘mmer’ which solves the mixed model equations proposed by Henderson [6], and it has been implemented to work directly with incidence and variance covariance matrices for each random effect. The function returns the variance components, the maximized log-likelihood, best linear unbiased estimators (BLUEs) for fixed effects and the BLUP solutions for random effects, along with other information of interest such as residuals, Akaike information criterion (AIC), Bayesian information criterion (BIC), etc.

In addition to the mixed model solver able to fit genome wide association (GWA) models in diploid and polyploidy organisms based on Yu et al. [29] and Rosyara et al. [30], the *sommer* package has been equipped with kernels to estimate additive relationship matrices based on Endelman [13] and VanRaden [27], and dominance and epistatic relationship matrices based on Su et al. [31] and Muñoz et al. [25], calculated respectively as:

$$A=\frac{ZZ\prime }{2\sum_{j=1}^{m}p_{j}(1-p_{j})}$$

$$D=\frac{ZZ\prime }{\sum_{j=1}^{m}2p_{j}q_{j}(1-p_{j}q_{j})}$$

$$\begin{array}{l} E_{aa}=A\#A\;\left(\text{additive by additive interactions}\right)\\ E_{dd}=D\#D\;\left(\text{dominance by dominance interactions}\right)\\ E_{ad}=A\#D\;\left(\text{additive by dominance interactions}\right) \end{array}$$

Denoting # the Hadamard product between matrices, **Z** being the scaled marker matrix, p and q the allelic frequencies for the j^th^ marker (j = 1…m). Markers are coded -1, 0, 1 with respect to a reference allele for the null homozygous, heterozygote, and positive homozygote respectively, for the additive relationship matrix **A**. On the other hand, markers are coded as 0 and 1’s for homozygotes and heterozygotes respectively for the dominance relationship matrix **D**.

Additional functions to 1) draw genetic maps, 2) convert letter format to numeric data, and 3) design matrices for half diallel designs, have been included in the package as well and are documented within the software.

### Genomic breeding value estimation in wheat

**Fig 1:**
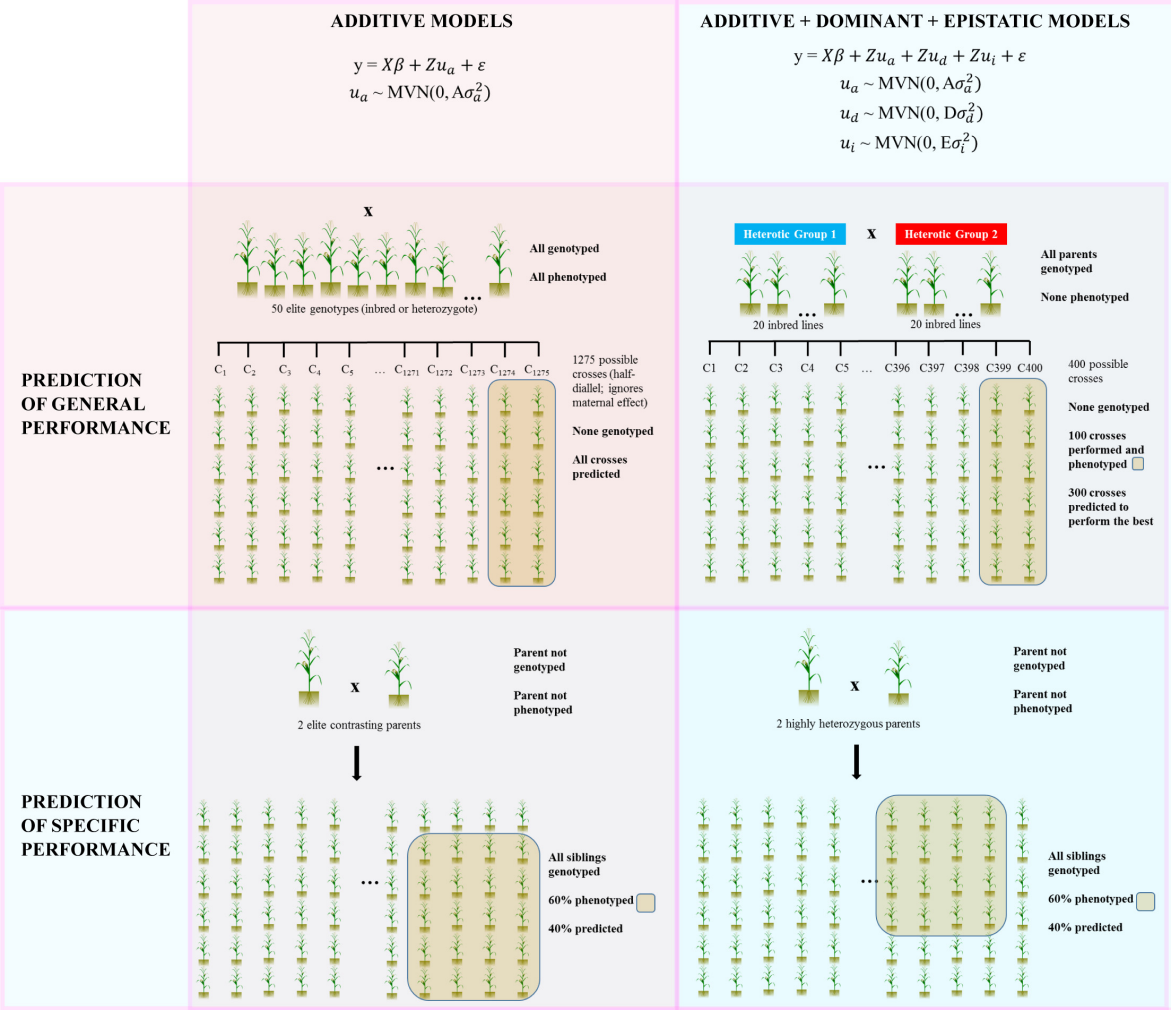
Examples of genome-assisted prediction performed using *sommer*. In the first row of the figure, genomic *prediction of general performance (cross performance prediction)* is summarized; in the 1^st^ square, the model to predict crosses with a single additive kernel (wheat example) is depicted. In the 2^nd^ square the prediction including additive, dominance and epistatic kernels is shown (single cross hybrid example). In the second row, genomic *prediction of specific performance (within cross performance prediction)* is shown; in the 1^st^ square, prediction within a biparental cross is shown using a single additive kernel for species displaying small or null heterotic effects whereas in the 2^nd^ square prediction within biparental populations is shown using additive, dominance and epistatic kernels (examples are included in the package).

Given that the realized genomic relationship matrix enters as a special case of the covariance structure for a random effect in a mixed model, and its incidence matrix represents the genotypes of such relationship matrix, the BLUPs of random effects are equal to the GEBV of the genotypes. In order to show the capabilities of the *sommer* package to predict the progeny performance when crossing lines without heterotic effects, such as in wheat lines, we used the dataset available in the BGLR package consisting in 599 lines which can be crossed hypothetically to produce 179,101 possible hybrids. We estimated the GEBV for the 599 lines for grain yield. Wheat, a self-pollinated crop, is usually bred by developing inbred lines by continuous cycles of selfing. Crosses among such lines usually do not display heterosis. This feature makes the prediction of a cross straight forward as the average among breeding values of the parental lines. On the other hand, predicting a maize cross which is known by being an outcross specie, heterosis needs to be taken into account. Therefore, is not possible to predict crosses by using the average breeding value among parental lines. Instead, independent general combining abilities are assumed for each parent and a specific combining ability is required to predict a maize cross (see [7,14] for a review of prediction in self pollinated and outcross species). In Fig 1 the two methods of kinship-based genomic prediction used in this investigation are shown. The model is easily fitted by specifying the variance-covariance structure **G** = **K**σ^2^_u_, with **K** being the additive or genomic relationship matrix (**A** or **A**_g_) among genotypes.

The BLUPs obtained by *sommer* were compared with rrBLUP [13], ASReml [21], regress [17], EMMREML [19], BGLR [16], and MCMCglmm [15]. We found all statistics such as BLUPs (u), BLUEs (βs), and variance components (σ^2^_s_) to be equal, showing that all software provided similar and sometimes identical results. Similar results were obtained using all the algorithms implemented in *sommer* (EMMA, EM, AI) to estimate variance components and other parameters. However, the EM and AI algorithms converged slower than EMMA when only one variance component was estimated due to the iterative procedure used with EM and AI. We found prediction accuracies of 0.51, 0.48, 0.38 and 0.46 for grain yield in the 4 mega-environments, respectively, which was consistent with the expected predictability upper bound expected for the prediction accuracy, which is the square root of heritability, h^2^ = 0.21 and $\sqrt{h^{2}}$ = 0.46 (Table 1) in the kinship-based prediction.

**Table 1:** Cross-validation of prediction accuracies using *sommer* (5-fold) for wheat and maize populations.

	Wheat^†^					Maize^†^			
	Env1	Env2	Env3	Env4	h^2^	A_(1)_	A_(2)_	A-D_(3)_	h^2^
Accuracy grain yield	0.51±.09	0.48±.10	0.38±.10	0.46±.09	0.21	0.18±0.14	0.21±0.16	0.37±0.16	0.18
Accuracy plant height	‡	‡	‡	‡	‡	0.41±0.12	0.43±0.13	0.68±0.06	0.62

Prediction accuracies for grain yield were obtained for each of the 4 mega environments available for the 599 lines of wheat. Prediction accuracies for grain yield and plant height were obtained for a maize population consisting of 100 hybrids tested in 4 locations using only additive (GCA) effects with a single variance component for both parents [A_(1)_], one variance component for each parent (GCA_1_ and GCA_2_; A_(2)_), and additive (GCA) and dominance (SCA) effects [A-D_(3)_].

† Accuracy values are averages over 500 runs of a 5-fold cross validation.

‡ Trait not evaluated in wheat.

### Single cross hybrid prediction in corn

One of the strengths of the *sommer* package is the ability to specify more than one random effect and their variance-covariance structure, which is usually necessary when genomic selection needs to be performed in species displaying heterotic effects. The model requires the estimation of GCA effects for parents and SCA effects for specific crosses. We used the simulated data for crosses between different heterotic groups that displayed heterotic effects for grain yield. We estimated the genomic relationship matrix (**A**_g_) for both groups of lines (**K**_1_ and **K**_2_) and calculated the Kronecker product of both matrices to obtain the SCA relationship matrix (**K**_3_), in order to predict the other 300 hybrids. We fitted the following model specifying the variance-covariance matrices with *sommer* and compared with ASReml [21], regress [17], EMMREML [19], BGLR [16] and MCMCglmm [15]. In sommer the model can be fitted in the following manner:

$$\begin{array}{l} library\left(sommer\right)\\ data\left(cornHybrid\right)\\ A=cornHybrid\$K\\ y=cornHybrid\$Yield\\ X1=model.matrix\left(∼Location,data=cornHybrid\right)\\ Z1=model.matrix\left(∼GCA1-1,data=cornHybrid\right)\\ Z2=model.matrix\left(∼GCA2-1,data=cornHybrid\right)\\ Z3=model.matrix\left(∼SCA-1,data=cornHybrid\right)\\ K1=A\left[levels\left(cornHybrid\$GCA1\right),levels\left(cornHybrid\$GCA1\right)\right]\\ K2=A\left[levels\left(cornHybrid\$GCA2\right),levels\left(cornHybrid\$GCA2\right)\right]\\ K3=kronecker\left(K1,K2\right)\\ ETA=list(GCA1=list\left(Z=Z1,K=K1\right),GCA2=list\left(Z=Z2,K=K2\right),SCA=list\left(Z=Z3,K=K3\right)\\ ans=mmer\left(y=y,Z=ETA,method=\text{AI}\right) \end{array}$$

or using a data frame

ans=mmer2(y∼1,random=∼GCA1+GCA2+SCA,G=list(GCA1=K2,GCA2=K2,SCA=K3),data=cornHybrid)

**Fig 2:**
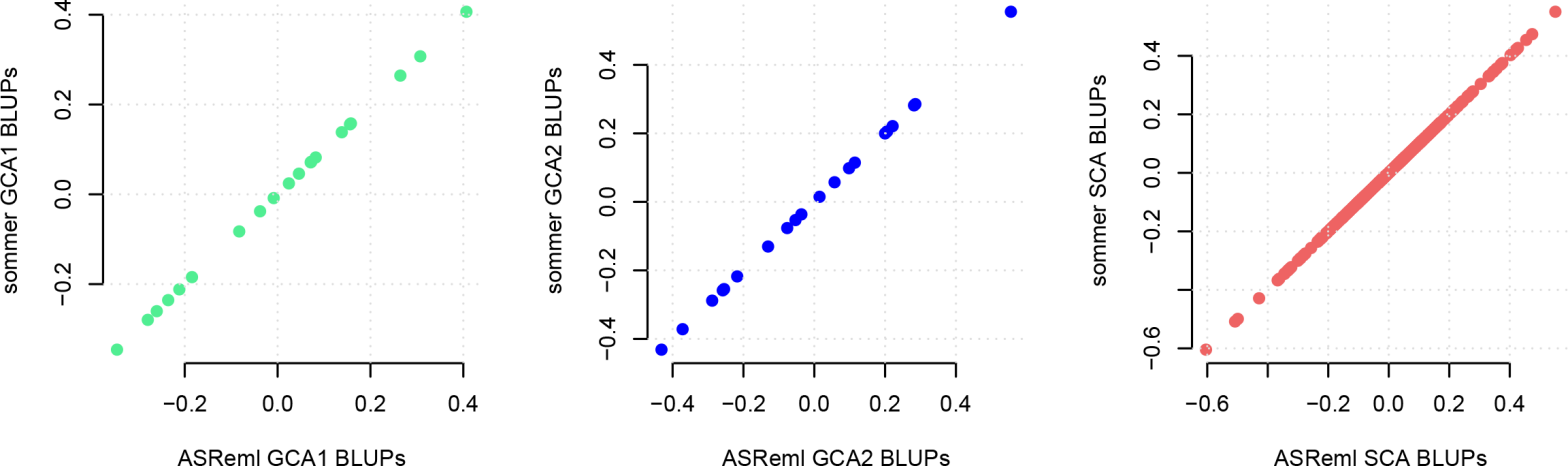
Best linear unbiased prediction (BLUP) comparisons for general and specific combining ability effects (GCA and SCA) using *sommer* versus ASReml. BLUPs for GCA and SCA related to grain yield were computed in a corn population with 400 individuals evaluated in 4 locations using *sommer* and ASReml. *Sommer* estimates are shown on the y axis and are similar to results from the ASReml estimates shown on the x axis.

The BLUPs for u_GCA1_, u_GCA2_, and u_SCA_ effects obtained by *sommer* and ASReml were the same (Fig 2) since they use by default the AI algorithm. On the other hand, *sommer* performed faster due to the use of the direct inversion AI algorithm compared to the mixed model equation based AI algorithm found in ASReml, based on the mathematical properties found by Lee et al. [22] when multiple random effects with dense covariance structures are present, and the use of an eigen decomposition on the **G** structure when there is a simple random effect [32] (please refer to the cites for more details on the differences between the two AI algorithms). Slight differences were found when comparing BLUPs from *sommer* with BGLR and MCMCglmm. However, this was expected due to the fact that Bayesian methods are based on Gibbs sampling, and require a high number of iterations to achieve the same parameters than likelihood software. In addition, *sommer*, regress and EMMREML were the fastest taking ~5 seconds to estimate the variance components and perform all calculations, but regress overestimated the error variance and returned some negative variance components, indicating that some constraints have not been implemented yet. EMMREML handles multiple random effects but does not return the variance components other than the first one. On the other hand, BGLR (iterations = 13000, burn-in = 2000) took 2 minutes and 2 seconds (*sommer* was 25 times faster), and MCMCglmm (iterations = 13000, default) took 7 minutes and 13 seconds (*sommer* was 87 times faster), showing that speed is one of the strengths of using REML-based software in dense genetic models.

**Fig 3:**
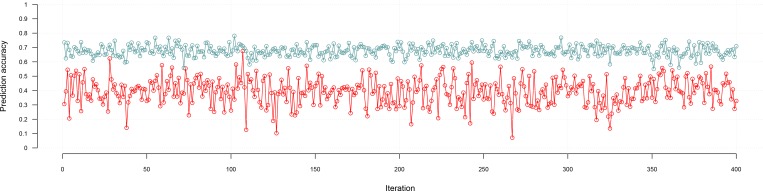
Prediction accuracy results using *sommer* in corn hybrids with a 5-fold cross validation. Cross validation results for plant height in a corn population of 400 individuals evaluated in 4 locations are shown in blue, whereas grain yield cross validation results for the same population is shown in red. Each dot represents the average of one run of a 5-fold cross validation.

Given that phenotypic data was masked for the corn hybrids, cross validation was conducted in order to assess the prediction accuracy of hybrids for plant height and grain yield using the hybrid prediction method stated above. The estimated heritability (h^2^) for grain yield was 0.18, and 0.62 for plant height in this population. According to selection theory, these values for grain yield set the upper bound for prediction to 0.43 for grain yield and 0.79 for plant height ($\sqrt{h^{2}}$). We found consistent results by using five-fold cross validations, which resulted in an average of 0.37±0.16 prediction accuracy for grain yield and 0.67±0.061 for plant height (Table 2; Fig 3). As expected, hybrids resulting from different heterotic groups were predicted taller and more productive than predicted hybrids resulting from a cross within the same heterotic group.

**Table 2:** Comparison of *sommer* versus the most common mixed model software available for genomic selection.

Feature	SAS	ASReml	lme4	rrBLUP	MCMC-glmm	BGLR	*sommer*	regress	EMM-REML
Open source			✓	✓	✓	✓	✓	✓	✓
Ability to specify var-cov structures for random effects	✓	✓	†	✓	✓	✓	✓	✓	✓
Estimation of more than one variance component	✓	✓	✓		✓	✓	✓	✓	✓
Basic expertise §	✓	✓	✓	✓	§	§	✓	✓	✓
Platform independent	✓	✓	✓	✓	✓	✓	✓	✓	✓
Ability to specify var-cov structures for residual structures	✓	✓			✓				
Use of sparse methods		✓	✓	‡	‡	‡	✓	‡	‡
Handles missing data	✓	✓	✓	✓	✓	✓	✓	✓	

Advantages and disadvantages of each software with check marks indicating whether they possess the stated feature.

† The *pedigreemm* package which is an extension of *lme4* allows the user to introduce pedigrees, but it does not allow the user to provide the variance-covariance matrices directly. Examples available in the *pedigreemm* package were run using *sommer* obtaining similar results, but *sommer* ran 4 times faster than *pedigreemm*. Examples are included in *sommer* documentation.

‡ Information not available.

§ These packages are based on Bayesian methods requiring the user to have a more advanced statistical background to decide the correct number of iterations, burn-in length and ability to analyze trace plots, and therefore the feature was defined as ‘Basic expertise’.

### Additive vs additive-dominance models

We found an increase in prediction ability when models fitted included additive and dominance effects (GCA+SCA) relative to a pure additive model (GCA) under the two assumptions that both parents have the same additive variance or each parent from a different heterotic group has its own additive variance. For grain yield the prediction accuracy changed from ~0.18–0.20 for the purely additive models to 0.52 in a model including GCA and SCA effects, whereas for plant height the prediction accuracy changed from 0.41–0.43 for the additive models to 0.85 for the model including additive and dominance effects (GCA+SCA; Table 2). This highlights the importance of considering dominance effects in addition to the only-additive models. The package provides kernels to calculate additive, dominance, and epistatic relationship matrices and examples of their use in the documentation, which have been shown to increase prediction accuracies in certain scenarios and family structures [31].

### Mixed model software availability and big data sets

There is limited open-source and user-friendly mixed models software that allow flexible specification of variance-covariance structures for random effects. We compared *sommer* and some of the most popular mixed model software available to highlight the strengths of this new software (Table 2). This software represents a valuable resource for genomic selection and GWAS studies, but it can also be used as any other mixed model software for analysis of non-plant and animal breeding experiments.

Although we have shown genomic selection applications using a kinship-based estimation of GEBV, some models depend on knowing marker effects, such as marker × environment interactions [33–35], which can be implemented as well using the *sommer* package. Half diallel designs and genome wide association studies (GWAS), and general mixed model analysis, can be performed in *sommer* as well, and examples are included in the documentation of the package.

A bigger data set with 5000 individuals genotyped with 10000 markers each, was simulated in order to test the performance of *sommer* against their counterparts under a bigger data set scenario with a single random effect. *Sommer* took 4 minutes to estimate all parameters for N = 5000 whereas, ASReml took 7.37 minutes, rrBLUP took 12.8 minutes, regress took 28.06 minutes, EMMREML 10.18 minutes, BGLR 21.5 minutes, and MCMCglmm 148 hours (using the default parameters specified in the M&M section. All calculations performed in a PC-Dell with processor 3.4 GHz Intel Core i7 and a 16 GB RAM memory). For a model with 3 variance components (GCA_1_, GCA_2_, SCA) with dense covariance structures consisting in 10578 hybrids *sommer* took 7.1 minutes to estimate all parameters, rrBLUP cannot estimate more than one variance component, regress took 31.13 minutes, ASReml took 114.56 minutes, EMMREML 15.61 minutes, BGLR took 289.4 minutes, and MCMCglmm took more than 12 days. This shows an important time reduction using *sommer* selected algorithms in comparison with other genomic prediction software in the magnitude of minutes for REML-based, and hours to days compared to Bayesian-based software (Table 3).

**Table 3:** Time comparison among different software for densest genomic models tested in the study.

No.Var.Comp.	Time	sommer	ASReml §	rrBLUP	regress	BGLR	MCMCglmm	EMMREML
One Var.	User	232.34	438.43	765.27	1679.88	1104.16	529527.09	610.85
Component	System	7.69	3.59	0.94	2.79	181.89	3715.65	0.18
N = 5000	Elapsed	240.04 #	442.73	766.43	1683.94	1291.71	533556.1	611.10
Three Var.	User	352.71	6860.85	‡	1858.99	11712.25	> 1058886 †	1130.92
Components	System	59.7	6.35	‡	3.53	5610.17	> 7431 †	4.89
N = 10585	Elapsed	425.96	6873.60	‡	1868.25	17364.27	> 1067112 †	1136.63 ¶

Time consumption for a GBLUP model with a single variance component (additive) with 5000 individuals and 10000 markers is shown in the first row. A GBLUP model with 3 variance components (additive, dominance, epistasis) with 10,585 hybrids to be predicted genotyped with 35,432 SNPs is displayed in the second row. The two models represent the biggest population sizes used in the study to highlight the differences when big data is encountered.

‡ No more than one variance component other that the error can be estimated.

† Work stopped after 12 days running the model.

§ In both models ASReml returned a warning message of abnormal termination.

¶ Although EMMREML can handle multiple random effects does not return the value for the variance components and cannot handle missing data.

# Using the average information with eigen decomposition proposed by Lee et al. [22].

**Fig 4:**
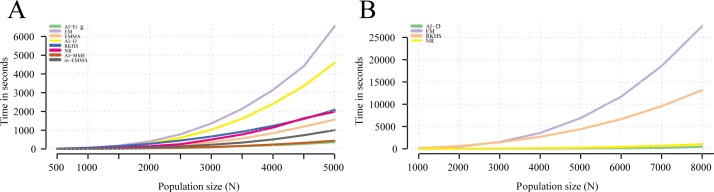
Time performance for different algorithms. In A) different color lines represent the different likelihood-based algorithms tested for populations sizes from 500 to 5000 in steps of 500 as a function of population size (N) for a single random effect. In B) the different color lines represent the algorithms able to deal with multiple random effects for a model with different population sizes (N), from 1000 to 8000 individuals for 3 random effects (GCA_1_, GCA_2_, and SCA).

We use the simulated dataset with 5000 individuals and 10000 markers to show how the computing time behaves as a function of the population size (N) for the different ML/REML algorithms found across software packages. We recorded the elapsed time for different population sizes from 500 to 5000 in intervals of 500. We found all algorithms to have a similar computation time for small population sizes (Fig 4A). As the problem increased in complexity (i.e. above 2000 individuals) we found the EMMA and AI algorithm using the eigen decomposition (AI-eig) to perform better than other algorithms such as EM and NR and AI-D. The time increment followed a quadratic behavior which exemplifies the issue of dealing with big populations (Fig 4B). The use of sparse methods such as the eigen decomposition on covariance matrices proposed by Lee and van der Werf [32] and Zhou and Stephens [36] constitute an important alternative to confront the population size increment that the researcher should take advantage of. On the other hand, when dealing with multiple random effects and more complex structures, we found the AI-D and NR algorithms to perform better than EM and RKHS sampling method.

The efficiency of *sommer* compared to most software relies on the use of the direct average algorithm (AI) proposed by Lee et al. [22], which surpasses in performance most algorithms when multiple random effects and dense covariance structures are present, the use the EMMA algorithm [20] or the use of an eigen decomposition in the **G** component when a single random effect is present [32], and the capability to switch to the expectation maximization algorithm (EM) [23] when covariance structures are rather sparse.

In addition, packages such as BGLR and regress require to form the kernel product **ZKZ**’ for each random effect as an input. This becomes an expensive operation as the model increase in size and complexity, although the Newton-Raphson algorithm performs quite fast, once this kernel has been formed. In the same way, packages such as MCMCglmm and ASReml-R require the inversion of the covariance matrix for the ‘ginverse’ argument, becoming quite computationally expensive as the covariance structures of the model increases in complexity. This was reflected in the computation time for the three random effects model, where inverting a 10,585 x 10,585 matrix (SCA matrix), corresponded to most of the computation time reported in Table 3 for those programs.

The most important strengths of the *sommer* package can be summarized as great flexibility to use different methods (algorithms), fast execution, and a friendly and intuitive interface that will help researchers to perform fast and easy genome wide association studies, and genomic selection strategies in research and breeding programs.

## Conclusion

The purpose of this paper was to describe and make available a general and flexible mixed model solver with popular and efficient algorithms in order to fit genomic selection models, genome wide association studies (GWAS) in diploid and polyploidy organisms, and other non-genetic analyses. Efficient mixed model association (EMMA), expectation-maximization (EM) and average information (AI) algorithms along with kernel methods for estimating additive, dominance and epistatic relationship matrices were developed and presented for plant breeders and scientists through the new R package *sommer*. At the core of the package, the ‘mmer’ function allows specification of flexible variance-covariance structures and can be used to solve marker-based and kinship-based versions of the genomic prediction and selection models. Examples using maize data illustrated the strengths of *sommer* to increase prediction accuracy in species displaying heterotic effects, which require the estimation of GCA and SCA effects with covariance structures for such random effects. We also showed the functionality of *sommer* in species with null or small heterotic effects and mainly additive effects by analyzing a dataset of wheat lines and obtaining similar results at a smaller computation time, and greater flexibility compared to those obtained with other popular software for genomic selection, based on Bayesian statistics and other REML-based software. Future implementations in *sommer* will include the addition of residual structures (R structures) to allow users to model spatio-temporal trends or longitudinal data, and other popular algorithms to provide more flexibility.

## Supporting Information

S1 File Code for analysis and tables. R code to recreate the analysis.
(R) Click here for additional data file.

## Abbreviations

AI: average information algorithm
BLUE: best linear unbiased estimator
BLUP: best linear unbiased predictor
EM: expectation maximization
EMMA: efficient mixed model association
GCA: general combining ability
GEBV: genomic-estimated breeding value
GV: genetic value
MAS: marker assisted selection
ML: maximum likelihood
NR: Newton-Raphson algorithm
QTL: quantitative trait loci
RAM: random access memory
REML: restricted maximum likelihood
SCA: specific combining ability
